# 

**Published:** 1896-05

**Authors:** 


### PERSONAL.

Dr. T. E. White, of Sedalia, Mo., has succeeded Dr. T. J. Turner, as State Veterinarian of Missouri. Dr. Turner's term of office expired in January.

Dr. R. C. Moore, of Holton, Kan., has taken the large practice which Dr. J. H. Wattles, of Kansas City, has been obliged to relinquish on account of failing health.

Dr. J. H. Wattles, Secretary of Kansas City Veterinary College and Professor of Anatomy, has resigned both positions on account of ill-health, and will go to southern Texas or Mexico, in the hope of finding relief and recovery in changed climatic conditions.

Veterinarian Charles A. Dohan is master of the Lima Hunt hounds.

Dr. W. B. E. Miller has been a very great sufferer from a prolonged and aggravated attack of rheumatism.

Dr. Geo. B. Rayner, of Philadelphia, is just recovering from a serious accident, caused by falling from a trolley car.

Dr. Leonard Pearson is nursing a very sore hand, the result of a bite from one of his canine patients, the wound having taken on an erysipelatous condition.

Dr. Wm. H. Lowe, of Paterson, N. J., was very active in looking after legislation objectionable to veterinarians in New Jersey.

Veterinarian Jos. M. Good, of Chattanooga, Tenn., is at present pursuing a course of study in human medicine at one of the Southern medical colleges.

Dr. John P. O'Leary, of Boston, Mass., graduate of the Veterinary Department of Harvard University, having passed the civil-service examination and received an appointment of Inspector under the Bureau of Animal Industry, has been assigned to Buffalo for duty.

At a special civil-service examination held in Kansas City, there were six applicants for meat-inspector, about forty for livestock-examiner, and ten for tagger, under the Bureau of Animal Industry.

Dr. B. F. Kaupp, of Nevada, Mo., and Dr. F. W. Hopkins, of

Fort Worth, Texas, were recently appointed meat-inspectors and assigned to Kansas City.

Dr. John Airth, of Sioux City, has been discharged from the meat-inspection service for cause.

Dr. Jas. A. Waugh has associated himself with Dr. E. I. Carter, of Pittsburg, and the infirmary maintained for a number of years by the latter will now be managed jointly by the new firm, as well as a union of their individual practices.

President Ridge, of the Pennsylvania State Veterinary Medical Association, has got right into harness. All his appointments of committees, county secretaries, delegates to sister associations for the year, besides to certain members the keeping of clinical notes of designated cases and remedies have been announced.

Veterinarian R. W. Hewitt, of Bridgeton, N. J., has been called to a place on the board of health of that city.

The Philadelphia Horse-Show will be held at the usual place on May 26th to 30th, inclusive. Veterinarians Pearson, Zuill, and Harger will officiate as Inspectors.

The Veterinary Department of the South Carolina Experiment Station, associated with the Clemson Agricultural College and under the direction of Dr. W. E. A. Wyman, covers in the college course instruction over a period of three years, the main stress being upon practical work. The board of trustees are about to erect an infirmary and purchase instruments, drugs, models, etc. Bulletins of a practical nature are issued from the experiment station and investigations conducted of outbreaks of contagious and infectious diseases among domesticated animals.

Veterinarian Hinman addressed the Manitoba Dairy Association on the use of tuberculin and the freedom from danger in its use as a diagnostic agent. Similar experiences were recorded by Drs. Rutherford and Thompson.

Dr. A. W. Clement, of Baltimore, Md., has recently been appointed State Veterinarian. This is a very creditable appointment, and we are sure will result in great good to the State. A graduate of Montreal Veterinary College, and for many years associated with the work of the Bureau of Animal Industry and in original work of investigation, the profession-at-large will expect much from him in his new sphere of work, in addition to the direct results given to his Commonwealth.

Dr. W. B. E. Miller, of Camden, N. J., a former alumni trustee of the American Veterinary College, has been elected a regular member of the board.

The alumni association of the American Veterinary College have selected as their representatives on the college board of trustees, subject to approval or rejection by the existing board, Drs. Herbert M. Lowe, of Paterson, N. J.; Chas. Burden, of New York City; and L. H. Howard, of Boston, Mass.

Dr. Alex. Glass, of Philadelphia, holds the arduous position of secretary to the Philadelphia Kennel-show.

The National Veterinary College had a class of sixteen students the past year. Of this number, eleven passed the final examination successfully and received the diploma of the college.

Pennsylvania's delegates at the annual meeting of the Veterinary Medical Association of New Jersey, Messrs. Ridge, Felton, and Lusson, were all present and thoroughly enjoyed the occasion. Their reception and the hospitality shown them was in keeping with the fame of that organization in this direction, and was well appreciated and will long be remembered. We trust that our sister association will be sure and delegate some of her members to visit the meetings in the Keystone State.

Dr. Hinman the newly appointed meat-inspector for Winnipeg, Manitoba, already realizes the difficulties surrounding his work, with every butcher having a private slaughter-house, and he suggests a central abattoir for his city, which will soon be the adopted plan of every large city that appreciates the value of meat-inspection properly executed.

Dr. Nelson P. Hinkley, of Buffalo, N. Y., has again been on the sick-list, and is now just able to be at his post of duty for a few hours daily. The JOURNAL trusts that his promised convalescence may be complete and speedy.

State Veterinarian Pearson, of Pennsylvania, finds his new duties absorbing most all of his time, and the work already accomplished in stamping out tuberculosis from our herds will result in great good to the communities where it has been accomplished, and from an educational point of view of value beyond one's power to estimate.

Dr. F. A. Nief, of California, has entered the national meat-inspection service, and is located at San Francisco.

Dr. W. E. B. Miller, of Camden, N. J., has been elected meat-inspector for the ensuing year, succeeding Dr. E. H. Landes, who formerly held the position.

Prof. R. S. Huidekoper has sufficiently recovered from his serious illness to contemplate a visit to Boston, Mass., where we hope his convalescence will be more rapidly attained.

Out of some nine applications filed for the position of consulting meat-inspector to the Department of Public Safety for Philadelphia, but four put in appearance before the Board at the appointed time.

